# Inhibition of Cyanobacterial Growth on a Municipal Wastewater Sidestream Is Impacted by Temperature

**DOI:** 10.1128/mSphere.00538-17

**Published:** 2018-02-28

**Authors:** Travis C. Korosh, Andrew Dutcher, Brian F. Pfleger, Katherine D. McMahon

**Affiliations:** a Department of Chemical and Biological Engineering, University of Wisconsin—Madison, Madison, Wisconsin, USA; b Environmental Chemistry and Technology Program, University of Wisconsin—Madison, Madison, Wisconsin, USA; c Department of Civil and Environmental Engineering, University of Wisconsin—Madison, Madison, Wisconsin, USA; d Microbiology Doctoral Training Program, University of Wisconsin—Madison, Madison, Wisconsin, USA; e Department of Bacteriology, University of Wisconsin—Madison, Madison, Wisconsin, USA; University of Iowa

**Keywords:** *Synechococcus* strain PCC 7002, cyanobacteria, nutrient removal, wastewater treatment

## Abstract

Sidestreams in wastewater treatment plants can serve as concentrated sources of nutrients (i.e., nitrogen and phosphorus) to support the growth of photosynthetic organisms that ultimately serve as feedstock for production of fuels and chemicals. However, other chemical characteristics of these streams may inhibit growth in unanticipated ways. Here, we evaluated the use of liquid recovered from municipal anaerobic digesters via gravity belt filtration as a nutrient source for growing the cyanobacterium *Synechococcus* sp. strain PCC 7002. The gravity belt filtrate (GBF) contained high levels of complex dissolved organic matter (DOM), which seemed to negatively influence cells. We investigated the impact of GBF on physiological parameters such as growth rate, membrane integrity, membrane composition, photosystem composition, and oxygen evolution from photosystem II. At 37°C, we observed an inverse correlation between GBF concentration and membrane integrity. Radical production was also detected upon exposure to GBF at 37°C. However, the dose-dependent relationship between the GBF concentration and the lack of membrane integrity was abolished at 27°C. Immediate resuspension of strains in high levels of GBF showed markedly reduced oxygen evolution rates relative to those seen with the control. Taken together, the data indicate that one mechanism responsible for GBF toxicity to *Synechococcus* is the interruption of photosynthetic electron flow and subsequent phenomena. We hypothesize that this is likely due to the presence of phenolic compounds within the DOM.

**IMPORTANCE** Cyanobacteria are viewed as promising platforms to produce fuels and/or high-value chemicals as part of so-called “biorefineries.” Their integration into wastewater treatment systems is particularly interesting because removal of the nitrogen and phosphorus in many wastewater streams is an expensive but necessary part of wastewater treatment. In this study, we evaluated strategies for cultivating *Synechococcus* sp. strain PCC 7002 on media comprised of two wastewater streams, i.e., treated secondary effluent supplemented with the liquid fraction extracted from sludge following anaerobic digestion. This strain is commonly used for metabolic engineering to produce a variety of valuable chemical products and product precursors (e.g., lactate). However, initial attempts to grow PCC 7002 under otherwise-standard conditions of light and temperature failed. We thus systematically evaluated alternative cultivation conditions and then used multiple methods to dissect the apparent toxicity of the media under standard cultivation conditions.

## Abstract (precis)

Cyanobacteria are viewed as promising platforms to produce fuels and/or high-value chemicals as part of so-called “biorefineries.” Their integration into wastewater treatment systems is particularly interesting because removal of the nitrogen and phosphorus in many wastewater streams is an expensive but necessary part of wastewater treatment. In this study, we evaluated strategies for cultivating *Synechococcus* sp. strain PCC 7002 on media comprised of two wastewater streams, i.e., treated secondary effluent supplemented with the liquid fraction extracted from sludge following anaerobic digestion. This strain is commonly used for metabolic engineering to produce a variety of valuable chemical products and product precursors (e.g., lactate). However, initial attempts to grow PCC 7002 under otherwise-standard conditions of light and temperature failed. We thus systematically evaluated alternative cultivation conditions and then used multiple methods to dissect the apparent toxicity of the media under standard cultivation conditions.

## INTRODUCTION

The need to develop non-petroleum-based platforms for fuel and chemical production is driving researchers to explore alternatives that harness renewable energy sources while minimizing other environmental impacts such as freshwater depletion, eutrophication, and the use of arable land for nonfood production. Cyanobacteria are particularly attractive platforms for such use due to their genetic tractability, high growth rates, halotolerance, and ability to be grown on nonproductive land with simple nutrient requirements (1, 2). According to published life cycle assessments, large portions of the associated costs of culturing photoautotrophs are tied to upstream costs, such as those associated with CO_2_ delivery and fertilizer application (3). High phosphorus-nitrogen removal rates and energy efficiencies have been reported for photobioreactor and open pond cultivation systems using wastewater streams rich in nitrogen and phosphorus (4, 5). Therefore, it may be possible to offset the requirement for fertilizer by reclaiming nutrients from wastewater. This approach could yield the sought-after non-petroleum-based alternative while also providing a more effective means of nutrient and metal removal than conventional wastewater treatment (6–8). Sidestreams from common wastewater treatment facilities such as supernatants or filtrates from processes performed for separation of solids are particularly promising nutrient sources, assuming that cyanobacterial strains can efficiently use them.

Of the many streams available in common wastewater facilities, the liquid fraction of anaerobic digestate is thought to be the most attractive nutrient source (8–11). Although digestate is rich in the inorganic constituents necessary for growth, it also contains dissolved organic matter (DOM), which has been shown to limit photosynthetic activity (12). DOM is a heterogenous mixture of aliphatic and aromatic compounds derived from the decomposition of living organisms (13). The chemical nature of wastewater-derived DOM is largely governed by the type of treated waste and the treatment process, but DOM is largely composed of hydrophilic, fulvic, and humic substances (14, 15). Humic substances can induce damaging permeability in model and bacterial membranes (16, 17). Various studies have also demonstrated that fulvic and humic acids can enhance the solubility of many organic compounds (18), which in turn would augment their bioavailability and potential membrane permeability. Many of these compounds are also photoreactive, producing toxic hydrogen peroxide and hydroxyl radicals (19, 20), which is of significant concern for phototroph cultivation.

The mode of DOM toxicity is thought to involve interactions with the protein-pigment complex of photosystem II (PSII) in photosynthetic organisms, although the exact molecular mechanism remains unclear (21). When the rate of light-induced damage to PSII exceeds its rate of repair, growth suppression and chlorosis result from the phenomenon known as photoinhibition (22). When damage caused by photoinhibition is sufficient to hamper the natural ability to consume electrons generated by photosynthesis, reactive oxygen species (ROS) are concomitantly produced as an undesired by-product. Prolonged oxidative stress halts protein translation through oxidation of specific cysteine residues in ribosomal elongation factors (23). Given these findings, it is increasingly evident that under conditions of sustained stress, regulation of electron flow is critical to maintain homeostasis in photosynthetic organisms (24, 25). Thus, it is important to understand the mechanisms by which DOM may be interrupting electron flow in order to capitalize on the potential of cyanobacteria to remediate wastewater and generate high-value chemicals.

In this study, we tested the practicality of using combined streams from a municipal wastewater plant as a nutrient source for cyanobacterial cultivation. We used the euryhaline cyanobacterium *Synechococcus* sp. strain PCC 7002 (PCC 7002) due to its exceptional tolerance of high light intensity, salt, and other environmental stresses (26, 27). Initial attempts to grow PCC 7002 in this nutrient source under standard environmental conditions for this strain (1% [vol/vol] CO_2_, a temperature of 37°C, and illumination of 200 µmol photons m^−2^·s^−1^) resulted in photobleached (white-yellow) cultures. In an effort to explain this observation while developing more-feasible cultivation conditions, we assessed the effects of many environmental parameters during PCC 7002 cultivation in wastewater-based media by monitoring changes in growth rate, photopigment abundance, oxygen evolution rates, membrane integrity, and membrane composition. High gravity belt filtrate (GBF) concentrations were associated with elevated DOM levels. Decreased photosynthetic oxygen production rates were also noted upon exposure to high levels of GBF. We observed marked membrane permeability, photosystem degradation, low growth rates, and ROS production in cultures exposed to GBF at 37°C. In comparison, the cultures grown at 27°C on GBF had reduced membrane permeability and robust growth rates as well as high levels of total fatty acids (FA) and an elevated level of unsaturated fatty acid content relative to the control. This suggests that the bioavailability of the photoinhibitory compound in GBF is governed by changes in membrane content and composition that occur during growth.

## RESULTS

### GBF and secondary effluent characteristics.

**FIG 1:**
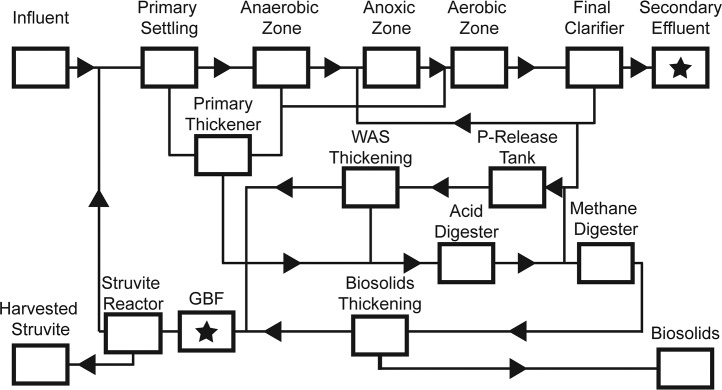
Flow diagram and nutrient streams obtained from the Nine Springs Wastewater Treatment Plant (Dane County, WI, USA). Stars indicate sampling points. WAS, waste activated sludge.

We measured concentrations of nutrients from the batches of GBF collected over the 6-month experimental period (Tables 1 and 2) to ascertain if it was a stable and reliable nutrient source for cultivating the cyanobacteria. Sampling points from the Nine Springs Wastewater Treatment Plant (Dane County, WI, USA) are shown in Fig. 1. In order to calculate nutrient stoichiometries, we took the sum of NH_3_-N and NO_3_-N to be representative of the bioavailable N. Nutrient levels were markedly more variable in the GBF than in the secondary effluent. The average molar ratio of bioavailable N to soluble reactive phosphorus (SRP) was 35 ± 7 in GBF (12.5% [vol/vol]) diluted with secondary effluent compared to 32 in medium A^+^.

**TABLE 1:** Nutrient composition of batch of 100% GBF used for subsequent experiments

Analyte^a^	Concn^b^ (mg·liter^−1^) ± SD
NH_3_-N	1,180 ± 135
NO_3_-N	7.5 ± 0.04
SRP	78 ± 10
COD	735 ± 4
TSS	467 ± 123
VSS	18 ± 2
TS	2,350 ± 36
TS (glass fiber filtered)	2,030 ± 42
TS (0.45-µm-pore-size membrane filtered)	2,000 ± 151

a SRP, soluble reactive phosphorus; COD, chemical oxygen demand; TSS, total suspended solids; VSS, volatile suspended solids; TS, total solids.

b Values shown represent means ± standard deviations (SD) of data from at least three technical replicates.

**TABLE 2:** Characteristics of GBF and secondary effluent over the 6-month experimental period

Source	Analyte	Concn^a^ (mg·liter^−1^) ± COV
GBF	NH_3_-N	920 ± 22%
	NO_3_-N	3.9 ± 43%
	SRP	54 ± 27%
	NH_3_-N	N.D.
		
Secondary effluent	NO_3_-N	19.3 ± 6.6%
	SRP	N.D.

a Values shown represent means ± coefficients of variation (COV) of data from at least three technical replicates. N.D., not determined.

**FIG 2:**
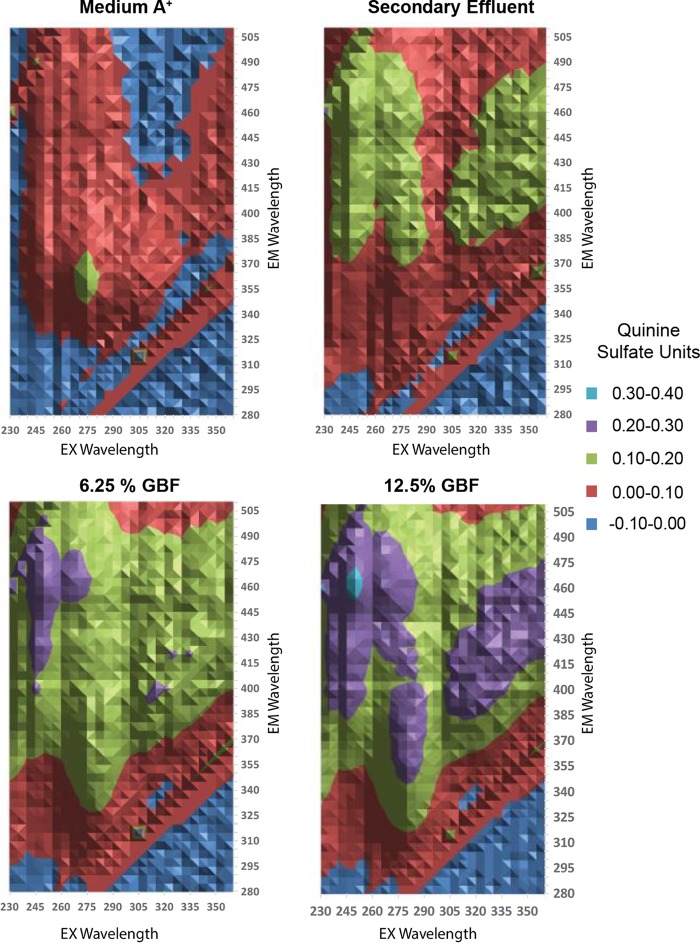
Excitation emission matrix of (A) medium A^+^, (B) secondary effluent, (C) 6.25% GBF, and (D) 12.5% GBF. Fluorescence values were normalized to 1 ppm of quinine sulfate–0.1 N H_2_SO_4_ at Ex/Em = 350/450 nm.

We also measured DOM quality in the secondary effluent and GBF using excitation (Ex)-emission (Em) matrix (EEM) fluorescence spectroscopy (28) because we hypothesized that DOM was linked to toxicity during cultivation, as had been shown in prior studies (16, 17, 29). EEM fluorescence spectroscopy revealed that the secondary effluent contained diffuse constituents, including humic acid-like spectra (excitation wavelengths of >280 nm and emission wavelengths of >380 nm) and fulvic acid-like spectra (excitation wavelengths of <250 nm and emission wavelengths of >350 nm) relative to medium A^+^ (Fig. 2). The distinction between the two substances has historically been based on solubility (30), but, compositionally, fulvic acids contain more acidic functional groups than humic acids (31). The fulvic acid content in preparations of media rose with increasing GBF concentrations. Additionally, at high concentrations of GBF, we detected absorbance in a distinct region (excitation wavelengths of 270 to 290 nm and emission wavelengths of 340 to 400 nm) that pointed to the presence of "soluble microbial products," which include aromatic amino acids, carbohydrates, or phenols (28, 32). Prior work has shown that the GBF stream of the Nine Springs Wastewater Treatment Plant contained high-molecular-weight DOM that differed in composition from other internal streams (33).

### Dose-dependent tolerance of GBF is a function of growth temperature.

**FIG 3:**
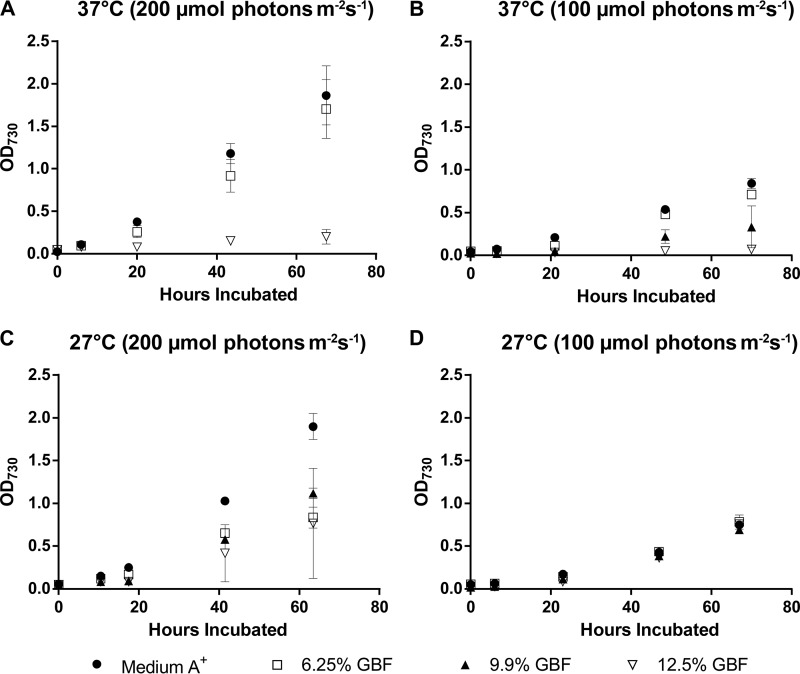
Biomass accumulation for cultures grown in medium A^+^ with 6.25%, 9.9%, or 12.5% (vol/vol) GBF media with 1% CO_2_ at (A) 37°C and 200 μmol photons m^−2^·s^−1^, (B) 37°C and 100 μmol photons m^−2^·s^−1^, (C) 27°C and 200 μmol photons m^−2^·s^−1^, or (D) 27°C and 100 μmol photons m^−2^·s^−1^. The values represent the means ± standard deviations (SD) of data from biological triplicates.

To evaluate the effects of GBF dosage on PCC 7002 physiology, we measured biomass accumulation (Fig. 3) and growth rates (Table 3) in media with GBF concentrations ranging from 6.25% to 12.5% (vol/vol) as a function of temperature (27°C versus 37°C) and light intensity (100 μmol photons m^−2^ s^−1^ versus 200 μmol photons m^−2^ s^−1^). Medium A^+^ served as a control. Higher temperatures depressed growth rates in GBF-based media. At 37°C, growth rates with 6.25% GBF at both 100 μmol photons m^−2^ s^−1^ and 200 μmol photons m^−2^ s^−1^ were most comparable to those seen with medium A^+^. Higher GBF concentrations had a more extreme effect on growth rates. However, this dose-dependent effect of GBF on growth rates was abolished when the cultivation temperature was lowered to 27°C. At 200 μmol photons m^−2^ s^−1^ and 27°C, GBF cultures grew half as quickly as the control and there was no significant difference between the tested GBF concentrations. At light intensities under 100 μmol photons m^−2^ s^−1^ and 27°C, growth rates were comparable across the medium conditions. Thus, successful cultivation using the more concentrated GBF media required adjusting both the light and temperature regimes.

**TABLE 3:** Growth rates with various temperatures and light intensities

Media	Light intensity (μmol photons m^−2^·s^−1^)	Temp (°C)	Growth rate ± SD (OD day^−1^)^a^
A^+^	200	37	0.66 ± 0.04
6.25% GBF	200	37	0.58 ± 0.05
12.5% GBF	200	37	0.05 ± 0.01
			
A^+^	100	37	0.28 ± 0.01
6.25% GBF	100	37	0.24 ± 0.01
9.9% GBF	100	37	0.11 ± 0.02
12.5% GBF	100	37	0.02 ± 0.00
			
A^+^	200	27	0.73 ± 0.08
6.25% GBF	200	27	0.32 ± 0.03
9.9% GBF	200	27	0.42 ± 0.06
12.5% GBF	200	27	0.28 ± 0.04
			
A^+^	100	27	0.25 ± 0.01
6.25% GBF	100	27	0.26 ± 0.02
9.9% GBF	100	27	0.24 ± 0.01
12.5% GBF	100	27	0.26 ± 0.02

a Data represent linear growth rates of cultures grown in medium A^+^ or GBF with the appropriate treatment under conditions of continuous illumination (200 μmol photons m^−2^·s^−1^ or 100 μmol photons m^−2^·s^−1^) with 1% CO_2_ at 37°C or 27°C. The values represent means ± SD of data from biological triplicates.

### Dose-dependent membrane permeability of GBF is a function of growth temperature.

**FIG 4:**
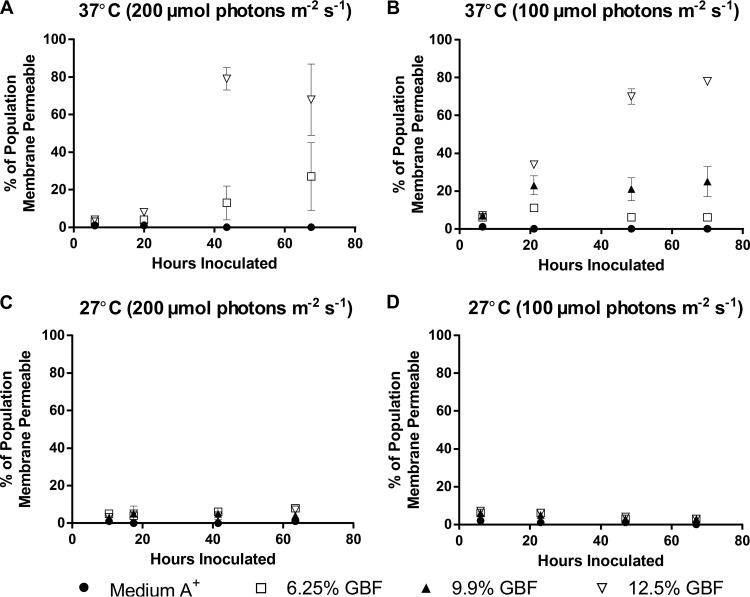
Membrane permeability of cultures grown in medium A^+^ with 6.25%, 9.9%, or 12.5% (vol/vol) GBF media with 1% CO_2_ at (A) 37°C and 200 μmol photons m^−2^·s^−1^, (B) 37°C and 100 μmol photons m^−2^·s^−1^, (C) 27°C and 200 μmol photons m^−2^·s^−1^, or (D) 27°C and 100 μmol photons m^−2^·s^−1^. The values represent means ± SD of data from biological triplicates.

We wondered whether the decreased growth rates in GBF at 37°C were a result of membrane permeability, given the known effect of humic acids on membrane integrity (16, 17). To track the dynamics of GBF-induced membrane permeability, we employed forward scatter flow cytometry using SYTO 59 as a counterstain to identify cells, which were subsequently visualized for membrane permeability using Sytox Green. As Sytox Green is a membrane-impermeative dye that fluoresces when binding to nucleic acids (34), fluorescence would indicate compromised outer membrane structures. Two distinct phases were identified upon exposure to GBF, which we interpreted as representing initial and chronic membrane permeability effects (Fig. 4). Initial membrane permeability was defined as the Sytox Green-positive events for samples analyzed within the first 10 h of growth, while chronic membrane permeability accounted for the Sytox Green-positive events at subsequent time points. As was the case for the growth rates, a relationship between both initial and chronic membrane permeabilities and increasing GBF concentrations was found at 37°C (Fig. 4A). While we still detected considerable initial membrane permeability with GBF exposure at 27°C, chronic membrane permeability decreased over time, mostly likely as a consequence of the increase in biomass (Fig. 3). Taking the results together, this suggested that there had been a temperature-dependent adaptation that ameliorated the susceptibility of cultures to GBF-induced membrane permeability.

### Exposure to GBF at high temperatures generates radicals and destroys the photosynthetic pigments.

**FIG 5:**
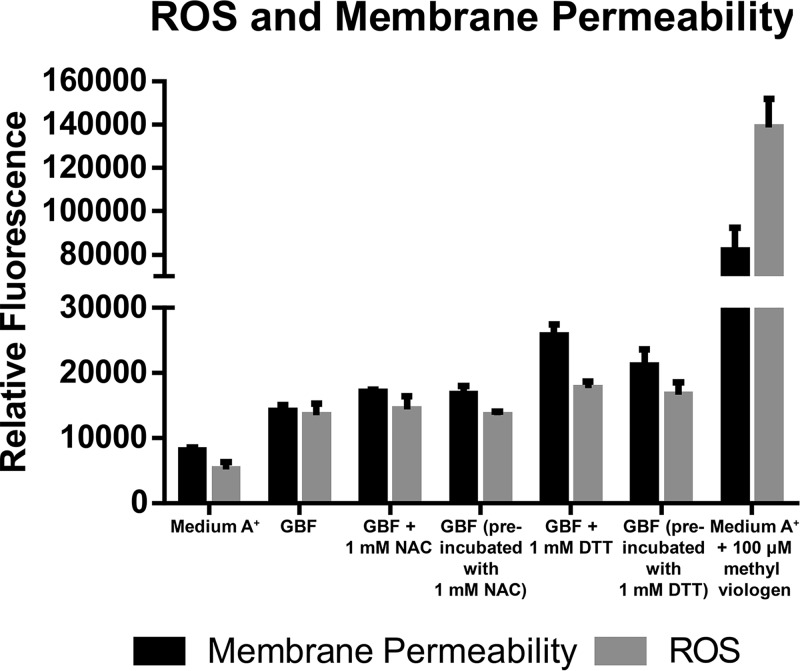
Reactive oxygen species and membrane permeability assay. Cells were grown to the early linear phase in medium A^+^ or 12.5% GBF with the appropriate treatment under conditions of continuous illumination (200 μmol photons m^−2^·s^−1^) with 1% CO_2_ at 37°C. Fluorescence values were normalized to OD_730_. The values represent means ± SD of data from biological triplicates.

We directly measured the ROS content and membrane permeability in response to overnight GBF exposure at 37°C using the fluorophores Sytox Green and CellROX Orange (Fig. 5). We examined the capacity of either of the two reducing agents dithiothreitol (DTT) and N-acetylcysteine (NAC) to quench the toxicity of the media, since they have antioxidant properties due to the direct reduction of disulfide bonds or as precursors for the antioxidant glutathione (35). To measure their effect on ROS production and culture viability after GBF exposure, we assessed ROS content and membrane integrity with either concurrent addition or preincubation of these quenching compounds in the diluted (12.5% [vol/vol]) GBF media. Addition of 100 µM methyl viologen to medium A^+^ served as a positive control. Exposure of cells to 12.5% GBF media resulted in marked ROS production and membrane permeability, which were not alleviated by thiol treatment.

**FIG 6:**
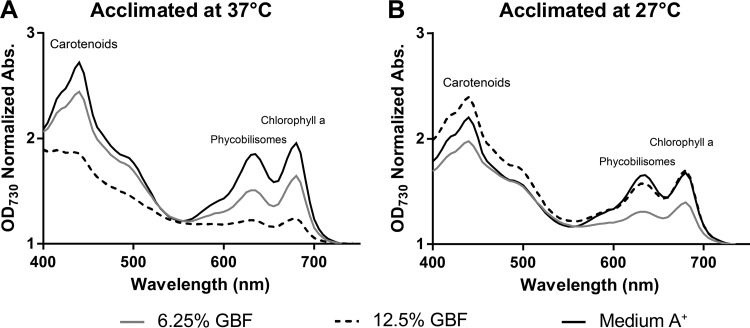
Absorption spectra of PCC 7002 cultures exposed to GBF at two different cultivation temperatures. Cultures were grown in medium A^+^ or 6.25% or 12.5% (vol/vol) GBF media under conditions of continuous illumination (200 μmol photons m^−2^·s^−1^) with 1% CO_2_ at (A) 37°C or (B) 27°C for 72 h. The spectra were recorded in dilute cell suspensions and normalized to OD_730_. The peak at 438 nm is due to carotenoids, the peak at 637 nm is due to phycobilisomes, and the peak at 683 nm is due to chlorophyll a.

Photobleaching of the photosynthetic pigments is also a common symptom of oxidative stress in photosynthetic organisms and is caused by the accumulation of ROS (36). To investigate the effects of GBF media on the photosynthetic pigmentation, we performed whole-cell absorbance scans of cultures cultivated at two different temperatures (27°C and 37°C) and at the same light intensity (200 μmol photons m^−2^ s^−1^). High GBF concentrations yielded enhanced chlorophyll (Chl), phycobilisome, and carotenoid degradation at 37°C (Fig. 6A). At 27°C, photosynthetic pigments maintained intact relative to the control, regardless of GBF concentration, implying less ROS production at this temperature (Fig. 6B).

### Exposure to GBF retards oxygen evolution.

**FIG 7:**
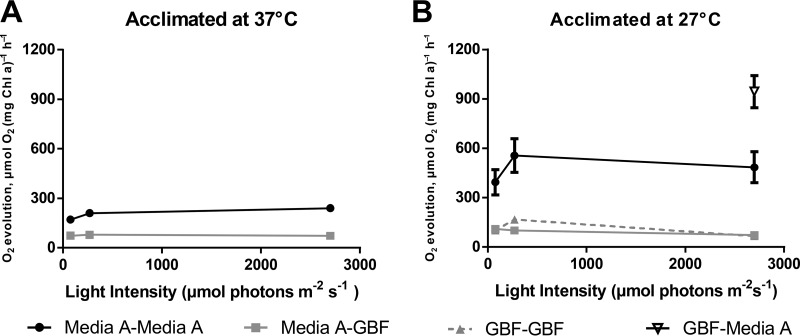
Rates of oxygen evolution as a function of acclimation temperature and light intensity. Cells were grown to the early linear phase in medium A^+^ or 12.5% GBF under conditions of continuous illumination (200 μmol photons m^−2^·s^−1^) with 1% CO_2_ at (A) 37°C or (B) 27°C. Cells were pelleted and resuspended in medium A^+^ or 12.5% GBF, and the rate of maximal oxygen evolution was measured with 10 mM HCO_3_^−^ as an electron acceptor at increasing light intensities. The values represent means ± standard errors (SE) of data from biological duplicates.

To better delineate the cause of initial toxicity associated with high GBF concentrations, we measured maximal oxygen evolution rates for strains briefly exposed to media with 12.5% (vol/vol) GBF while increasing the light intensity. Measurements of levels of photosynthetic oxygen evolution would allow an indirect assessment of PSII activity and electron transfer (37). Cultures were grown to the early linear phase in medium A^+^ or GBF media, washed, and resuspended in the appropriate media. Resuspension medium was saturated with HCO_3_^−^ (10 mM) in order to prevent inorganic carbon limitation. As expected, cells grown and assayed in medium A^+^ at 37°C showed a clear increase in the O_2_ evolution rate as the light intensity approached saturation at 2,700 μm photons m^−2^ s^−1^, reaching a maximal rate of 240 ± 8 µmol O_2_ (mg chlorophyll a)^−1^ h^−1^ (Fig. 7A). Cells grown in medium A^+^ at 37°C but assayed in 12.5% GBF had diminished O_2_ evolution rates at all light intensities, plateauing with a rate of 79 ± 6 µmol O_2_ (mg Chl a)^−1^ h^−1^ at an intensity of 270 μmol photons m^−2^ s^−1^ (Fig. 7A). Thus, exposure to GBF under these conditions caused an immediate decrease in O_2_ production.

Next, we examined the effect of temperature in a similar experiment. Assays carried out in medium A^+^ after growth in medium A^+^ at 27°C showed much higher maximal O_2_ evolution rates at all tested light intensities than those seen with cells grown at 37°C (Fig. 7B), with the higher rates peaking at 556 ± 102 µmol O_2_ (mg Chl a)^−1^ h^−1^. This was expected because elevated O_2_ evolution rates in cells grown at low temperatures have been previously reported and were attributed to a substantial change in photosystem stoichiometry (38). The O_2_ evolution rates of cells grown in medium A^+^ at 27°C and then resuspended in 12.5% GBF stayed relatively constant at all of the tested intensities and were roughly 5-fold lower than those seen in the controls, with a maximal rate of 109 ± 22 µmol O_2_ (mg Chl a)^−1^ h^−1^ at an intensity of 75 μmol photons m^−2^ s^−1^. We compared the rates described above to those from cultures grown in 12.5% GBF at 27°C to test if adaptation to GBF was met with changes in photosynthetic activity. At the examined light intensities, O_2_ evolution rates with 27°C GBF-adapted cultures were not statistically different from those measured in cultures grown at the same temperature in medium A^+^. Finally, we conducted the inverse experiment, using cultures grown in GBF media at 27°C but assayed in medium A^+^ under conditions of saturating light. Interestingly, those cultures displayed the highest evolution rate seen under any of the tested conditions, at 944 ± 96 µmol O_2_ (mg Chl a)^−1^ h^−1^ (Fig. 7B). This suggested that there is a period of dynamic photosynthetic adaptation to overcome the stress of GBF and that, when the stress is removed, the cells have an enhanced capacity for photosynthetic activity.

**FIG 8:**
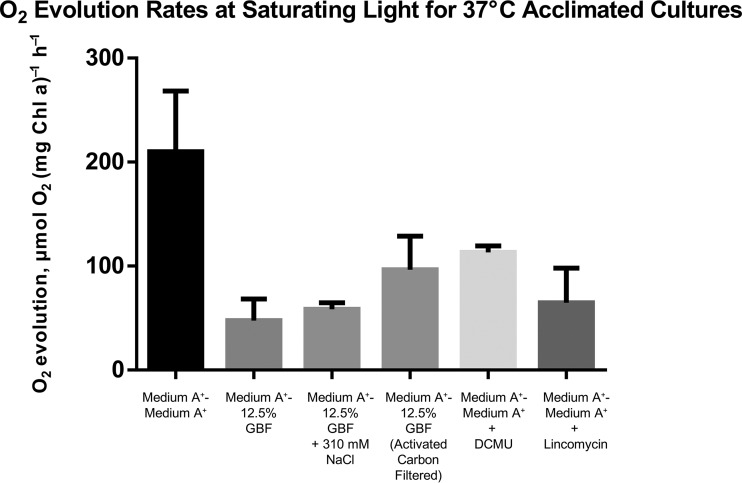
Photosynthetic oxygen evolution rates under saturating light conditions. Cells were grown to the early linear phase in medium A^+^ under conditions of continuous illumination (200 μmol photons m^−2^·s^−1^) with 1% CO_2_ at 37°C. Cells were pelleted and resuspended in medium A^+^, 12.5% GBF, 12.5% GBF plus 310 mM NaCl (control for osmolarity), 12.5% GBF prefiltered with activated carbon, or medium A^+^ ± 10 µM DCMU (control for inhibition for electron transport) or were pretreated with 800 µg·ml^−1^ lincomycin for 1 h (control for the inhibition of protein synthesis). Maximal oxygen evolution rates were measured with 10 mM HCO_3_ as an electron acceptor at 2,700 μmol photons m^−2^·s^−1^. The values represent means ± SE of data from biological duplicates.

Additional assays were performed under conditions of light saturation (2,700 μmol m^−2^ s^−1^) with cultures adapted to 37°C (Fig. 8) to further examine the effects of the medium conditions on oxygen evolution rates. Again, the oxygen evolution rates in GBF (73 ± 8 µmol O_2_ [mg Chl a]^−1^ h^−1^) were much lower than those seen with medium A^+^ (248 ± 21 µmol O_2_ [mg Chl a]^−1^ h^−1^). In order to rule out the effect of differences in overall ionic strength, we increased the osmolarity of the GBF medium by adding NaCl to match the levels found in medium A^+^ and found no statistically significant difference in oxygen evolution compared to the results seen with experiments performed without added NaCl. Attempts to eliminate the inhibitory effect of GBF on oxygen evolution by gravity filtration through powdered activated carbon were unsuccessful. We also measured oxygen evolution rates in control experiments designed to inhibit photosynthetic electron transfer through addition of 10 µM DCMU [3-(3,4-dichlorophenyl)-1,1-dimethylurea] to medium A^+^ (39) and to prevent *de novo* protein synthesis by pretreating cultures with 800 µg·ml^−1^ lincomycin in medium A^+^ (40), prior to incubation under conditions of light saturation for 1 h. However, the evolution rates determined with these controls were higher than those seen under conditions of treatment with GBF. Therefore, no definitive conclusion about the molecular mechanism responsible for reduced oxygen evolution rates in the presence of GBF could be made.

### Acclimation to GBF changes lipid content and composition.

On the basis of the results described above, we hypothesized that the temperature-dependent adaptation may be related to changes in membrane content and composition. We extracted total fatty acids of cultures grown at 27°C for 72 h in 12.5% GBF or medium A^+^ and analyzed the content after derivatization. We could detect and resolve all major saturated and unsaturated fatty acid species (Table 4). Cultures grown in GBF had greater totals of assayed fatty acid species (27 ± 7 mg fatty acid methyl ester [FAME] grams of dry cell weight [gDCW^−1^]) compared to cells grown in medium A^+^ (15 ± 1 mg FAME gDCW^−1^) (Table 4). The most abundant fatty acid in all samples was 16:0 (42% to 45% of the total fatty acids). C18:2 Δ9,12 fatty acids comprised a significant fraction of medium A^+^-grown cultures, with 22% of the total fatty acid species. However, in GBF-grown cells, C18:2 Δ9,12 fatty acids composed only 15% of the total fatty acid pool, while the levels of C18:3 Δ9,12,15 fatty acids were twice as high in GBF-grown cells (16%) as they were in medium A^+^-grown cells (9%) (Table 4). These results suggest that the cells grown in GBF were altering their membrane homeostasis compared to the standard growth seen in medium A^+^. While we did not differentiate between the outer membranes, plasma membranes, or thylakoid membranes with respect to the levels of fatty acid content and composition characteristics in our study, prior work done with the cyanobacterium *Synechocystis* PCC 6803 found similar fatty acid composition characteristics in comparisons between thylakoid and plasma membranes (41).

**TABLE 4:** Fatty acid content and composition of cultures grown in medium A^+^ or 12.5% GBF media under conditions of continuous illumination (200 μmol photons m^−2^·s^−1^) at 27°C for 72 h

Growth condition^a^	mg FA gDCW^−1^ (% FA) for fatty acid species^b^:					
	C16:0	C16:1 Δ9	C18:0	C18:1 Δ9	C18:2 Δ9,12	C18:3 Δ9,12,15
Medium A^+^	6.7 ± 0.7 (44 ± 1)	2.0 ± 0.1 (13 ± 1)	0.2 ± 0.1 (2 ± 0)	1.5 ± 0.2 (10 ± 0)	3.3 ± 0.2 (22 ± 1)	1.4 ± 0.1 (9 ± 1)
						
GBF	11.6 ± 3.1 (43 ± 0)	3.5 ± 0.8 (13 ± 0)	1.3 ± 0.3 (5 ± 0*)	2.2 ± 0.7 (8 ± 0)	3.9 ± 0.8 (15 ± 1*)	4.4 ± 1.6 (16 ± 1*)

a Strains were grown in medium A^+^ or 12.5% GBF media under conditions of continuous illumination (200 μmol photons m^−2^·s^−1^) at 27°C for 72 h. Fatty acids were extracted and derivatized as previously described. The values represent means ± SD of data from two independent experiments.

b *, data represent statistically significant differences from control results (*P* value, <0.005 [unpaired *t* test]).

## DISCUSSION

The amount and distribution of arable land and potable water are projected to change over the next several decades due to climate change, while increases in the global population and standards of living are expected to increase demand for these resources (42). Microalgal cultivation integrated with industrial and municipal wastewater treatment circumvents many of the resource concerns raised over biofuel production (2) while simultaneously removing additional nutrients and pollutants present in the wastewater (43). *Synechococcus* sp. strain PCC 7002 is an intriguing possible platform for this purpose, since it can be readily engineered to produce high-value chemicals (44). Under standard environmental conditions, however, we were unable to obtain robust growth of PCC 7002 using a diluted municipal sidestream (GBF) as a nutrient source. We hypothesized that this effect may have been due to the presence of DOM, which has been demonstrated to cause a decrease in photosynthetic performance in various cyanobacterial strains (5–47). We investigated the effects of light intensity and temperature on the physiology of cultures grown in GBF in efforts to find conditions conducive to high growth rates and biomass generation and to better understand the mechanisms of GBF toxicity.

We propose that the herbicidal effect of GBF is primarily due to PSII inhibition, as shown in Fig. 7 and 8, brought about by the presence of quinone or phenolic compounds within the soluble microbial products found in our EEM scans (Fig. 2). Humic material enriched in quinone and phenolic compounds can inhibit cyanobacterial growth (48, 49). Phenolic photosynthetic electron transfer inhibitors, such as 2,5-dibromo-3-methyl-6-isopropyl-p-benzoquinone (DBMIB), alter the redox potential of the plastoquinone (PQ) pool of PSII by blocking forward electron transfer to the cytochrome *b*_6_/f complex (50). The immediate decrease in oxygen evolution (Fig. 7) and the high rates of initial membrane permeability (Fig. 4) upon GBF exposure suggest that the toxic compound(s) rapidly crosses the outer membrane, where it subsequently interrupts photosynthetic electron flow. Chronic exposure to phenolic herbicides eventually leads to radical-catalyzed back reactions that trigger ROS formation (51) (Fig. 5) that facilitates complex destruction (Fig. 6) and cell death (6, 52–55). Some phenolic herbicides may also act as arylating agents, causing covalent binding to macromolecules via Michael addition and thiol pool depletion (56). The inability of the reducing agents that we tested to maintain membrane integrity suggests that GBF-induced cytomembrane permeability is likely caused by redox cycling and not arylation.

We found that cultivation temperature was an important factor that determined whether PCC 7002 could grow under conditions of high GBF concentrations. Numerous physiological processes are altered at low temperatures (57). Notably, the fatty acyl chains in membranes undergo a transition from a fluid state to a nonfluid state (58). Cyanobacterial membrane organization is complicated by the simultaneous existence of the outer, plasma, and thylakoid membranes, each with a designated physiological function (59). As part of the homeoviscous response to a shift to a lower temperature, cyanobacteria alter the expression of desaturases in their plasma and thylakoid membranes (60) to increase unsaturated fatty acid content and maintain optimal membrane function (61). Optimal thylakoid membrane fluidity is a critical factor in photosynthetic electron transport, because coutilized redox components must be able to move quickly between photosynthetic and respiratory complexes to ensure ideal electron flow (62, 63). Temperature is known to influence the kinetics governing the redox state of plastoquinone (PQ) through alterations in thylakoid membrane composition and fluidity (64). We hypothesize that alteration of the thylakoid membrane to circumvent GBF-induced changes in the overall redox state is also an important component of the adaptive response to lower temperatures, given the increase in the total levels of unsaturated fatty acids observed during cultivation in GBF-based media (Table 4). Upon shifts to a lower temperature, PCC 7002 has also been shown to translocate the phycobilisomes from PSII to PSI (65), thereby decreasing the ratio of reducing power to proton motive force (66). Mutant cells of the cyanobacterium *Synechocystis* sp. strain PCC 6803 that lacked polyunsaturated fatty acids were unable to perform these state transitions at low temperatures (67). This change in the photosystem arrangement at 27°C may also contribute to acclimation to the photoinhibitory compound. Future efforts to increase tolerance of these toxicants might include disruption of the inhibitor binding sites (68, 69) or optimization of thylakoid membrane fluidity (70).

### Conclusions.

Under standard cultivation conditions of 37°C with the cyanobacterium *Synechococcus* sp. strain PCC 7002, there was a dose-dependent relationship between the liquid anaerobic digestate (GBF) concentration and membrane permeability. This dose-dependent relationship mirrored ROS production and photopigment degradation. The digestate contained dissolved organic matter constituents that were likely affecting photosynthetic electron transport. Decreasing the cultivation temperature to 27°C enabled robust cultivation at high digestate concentrations, resulting in high levels of biomass productivity. This temperature-dependent tolerance may be due to changes in membrane properties. Our report highlights an unanticipated influence of dissolved organic matter on photosynthetic growth and physiology in wastewater-based media, as well as a potential mechanism for tolerance in low temperatures.

## MATERIALS AND METHODS

### Media and growth conditions.

*Synechococcus* sp. strain PCC 7002 was obtained from the Pasteur Culture Collection of Cyanobacteria. Experiments were performed with a strain of PCC 7002 harboring the *aaC1* gene encoding a gentamicin resistance marker in the A2842 locus (*glpK*) to maintain axenic cultures. Strains were grown and maintained on solid medium composed of medium A^+^ (71) supplemented with 5 µM NiSO_4_ (72) with 1.5% Bacto agar. Strains were cultured from a single colony in 250-ml baffled flasks with 50 ml media with 1% CO_2_-enriched air at 150 rpm in a Kuhner ISF1-X orbital shaker. Temperature was maintained at 37°C or 27°C, and light intensity was fixed at approximately 200 µmol photons m^−2^·s^−1^ or 100 µmol photons m^−2^·s^−1^ via the use of a custom light-emitting-diode (LED) panel. Strains were preacclimated to the culture conditions overnight before being inoculated into fresh media. Optical density at 730 nm was measured in a Tecan M1000 plate reader.

Wastewater-derived medium was obtained from the Nine Springs Wastewater Treatment Plant (Dane County, WI, USA). The plant is configured for biological nutrient removal via a modified University of Cape Town process with no internal nitrate recycling and stable performance, yielding high secondary effluent quality (total phosphorus, <1 mg P/liter; total ammonia, <1 mg N/liter; total nitrate, ~15 mg N/liter) (73). Anaerobic digesters are used for stabilization of solids, and the resulting digested material is passed over a gravity belt filter for dewatering. The system includes an Ostara WASSTRIP process to recover phosphorus. The filtrate (GBF) served as the primary source of phosphorus and reduced nitrogen for our cultures, and effluent from the postmainstream secondary treatment clarifier (secondary effluent) served as a diluent. GBF was gravity filtered through a paper coffee filter to remove any exceptionally large flocs and was then stored at −80°C until use. Secondary effluent was collected 1 to 4 days before each experiment and held under conditions of refrigeration at approximately 2°C. Experimental medium was composed primarily of secondary clarifier effluent and GBF, combined in different proportions. Unless otherwise noted, GBF was used at a concentration of 12.5% (vol/vol) in secondary effluent, supplemented with trace metals and vitamin B12 at the concentrations found in medium A^+^, as well as KH_2_PO_4_ at a molar ratio of 1:32 soluble reactive phosphorus (SRP) to bioavailable nitrogen (the sum of NH_4_^+^ and NO_3_^−^). All medium was buffered with Tris-HCl and adjusted to pH 8.0 with potassium hydroxide or hydrochloric acid before sterilization by autoclaving was performed, and gentamicin was added at working concentrations (30 μg/ml) after cooling.

### Staining, flow cytometry, and fluorescence measurements.

The membrane permeability of cyanobacterial cells was recorded by a flow cytometer (FACSCalibur; BD Biosciences, San Jose, CA). After growth in the respective media, cells were centrifuged (2 min, 5,000 relative centrifugal force [RCF]), decanted, and resuspended in 1 ml of Tris-buffered saline (TBS) solution (pH 8.0). To identify membrane-compromised cells, Sytox Green (Life Technologies, Inc.) was also added to each sample (1 μM). SYTO 59 (Life Technologies, Inc.) was added to each sample (1 μM) as a nucleic acid counterstain. As a control for a permeabilized membrane, cells were resuspended in 190-proof ethanol. Sytox green fluorescence was visualized using 488-nm-wavelength laser excitation, and the emission area was read using a 530/30-nm-wavelength bandpass filter. A 633-nm-wavelength laser coupled with a 661/16-nm-wavelength bandpass filter was used for SYTO 59 visualization. Analysis of the cytometric data was carried out with CellQuest Pro software (BD Biosciences, San Jose, CA, USA).

To assess reactive oxygen species production, cells (optical density at 730 nm [OD_730_] of 1) were incubated overnight in a reaction mixture consisting of medium A^+^ or 12.5% (vol/vol) GBF or 12.5% (vol/vol) GBF plus 1 mM dithiothreitol (DTT) or 12.5% (vol/vol) GBF plus 1 mM N-acetylcysteine (NAC) or (as a positive control) medium A^+^ ± 100 µM methyl viologen at 37°C with 5% CO_2_ at a light intensity of 200 µmol photons m^−2^·s^−1^. Cells were washed in TBS, and either Sytox Green (1 μM) or CellROX Orange reagent (Life Technologies, Inc.) (5 μM) was added. After 30 min of incubation in darkness at 37°C, fluorescence was measured (excitation wavelength/emission wavelength [Ex/Em], 545/565 nm for CellROX Orange) and (Ex/Em, 504/523 nm for Sytox Green) in a Tecan M1000 plate reader.

### GBF characterization.

SRP, ammonia, nitrate, and nitrite concentrations were determined for all secondary clarifier effluent and GBF samples used in these experiments. In addition, the GBF was tested for total suspended solids (TSS), volatile suspended solids (VSS), total solids (TS), and chemical oxygen demand (COD). Ammonia, SRP, and COD concentrations were determined by colorimetric tests using reagents from Hach. Nitrate and nitrite concentrations were determined using high-performance liquid chromatography (Shimadzu) with a C_18_ column and photodiode array detector (74). TSS, VSS, and TS were measured according to standard methods 2540 D, 2540 E, and 2540 B, respectively, with 47-mm-diameter glass fiber filters (Whatman) used for TSS and VSS (75). Fluorescence EEM measurements and UV light-visible light (UV-Vis) absorbance scans were conducted using an M1000 Tecan plate reader and a UV-transparent plate (Costar 3635). Fluorescence intensity was normalized to quinine sulfate units (QSU), where 1 QSU represents the maximum fluorescence intensity of 1 ppm of quinine sulfate in 0.1 N H_2_SO_4_ at Ex/Em = 350/450. Rayleigh scatter effects were removed from the data set.

### Biochemical analyses.

Batch cultures were further assayed for dry cell weight (DCW), fatty acid content, oxygen evolution rates, and chlorophyll a content. Cells were concentrated by centrifugation, washed in TBS, and lyophilized overnight to obtain DCW. Fatty acids from approximately 10 mg of DCW with 10 mg·ml^−1^ pentadecanoic acid as an internal standard were converted to methyl esters, extracted with n-hexane, and subjected to gas chromatography-flame ionization detector (GC-FID) analysis on a Restek Stabilwax column (60 m, 0.53-mm inner diameter [ID], 0.50 µm) (76). Photosynthetic oxygen evolution from whole cells was measured with a Unisense MicroOptode oxygen electrode with 10 mM NaHCO_3_ that was illuminated with a slide projector at photosynthetic photon flux densities ranging from 76 to 2,700 μmol photons m^−2^ s^−1^ for 30 min at room temperature (77). Cells were collected by centrifugation and resuspended in the appropriate media to give an OD_730_ of 1.0. Chlorophyll a measurements were done via a 100% chilled methanol extraction procedure (78). Chlorophyll a levels were calculated via the following equation: Chl_a_ = 16.29 × A^665^ − 8.54 × A^652^ (79).
